# A Case of Hypogonadotropic Hypogonadism Caused by Opioid Treatment for Nonmalignant Chronic Pain

**DOI:** 10.1155/2012/740603

**Published:** 2012-12-25

**Authors:** Yukiko Tabuchi, Tetsuyuki Yasuda, Hideaki Kaneto, Tetsuhiro Kitamura, Junji Kozawa, Michio Otsuki, Akihisa Imagawa, Aya Nakae, Youichi Matsuda, Hironobu Uematsu, Takashi Mashimo, Masahiko Shibata, Iichiro Shimomura

**Affiliations:** ^1^Department of Metabolic Medicine, Graduate School of Medicine, Osaka University, Suita 565-0871, Japan; ^2^Anesthesiology and Intensive Care, Graduate School of Medicine, Osaka University, Suita 565-0871, Japan; ^3^Department of Anesthesiology, Osaka Prefectural Medical Center for Respiratory and Allergic Diseases, Habikino 583-8588, Japan; ^4^Pain Medicine, Graduate School of Medicine, Osaka University, Suita 565-0871, Japan

## Abstract

We report a case of 42-year-old male patient with hypogonadotropic hypogonadism. He suffered from general fatigue and erectile dysfunction after the treatment with transdermal fentanyl for chronic pain by traffic injury. Endocrine examinations and hormone stimulating tests showed that he had hypogonadotropic hypogonadism. Brain magnetic resonance imaging (MRI) showed no abnormal findings, and he had no past history of accounting for acquired hypogonadotropic hypogonadism. Therefore, his hypogonadism was diagnosed to be caused by opioid treatment. Although opioid-induced endocrine dysfunctions are not widely recognized, this case suggests that we should consider the possibility of endocrine dysfunctions in patients with opioid treatment.

## 1. Introduction

Opioids have been widely used for the management of acute, chronic, malignant, and nonmalignant pain in the world [1]. However, opioids have several adverse effects such as itching, nausea, constipation, and respiratory suppression. Recently, it has been reported that chronic use of opioids induces endocrine dysfunctions in humans [2–11]. The most common endocrine dysfunction is hypogonadism leading to not only a decrease in sexual function but also impaired physical and psychological conditions such as fatigue, muscle weakness, osteoporosis, and emotional disturbances [2–9]. On the other hand, it has been reported in a small number of cases that adrenal insufficiency and adult growth hormone deficiency can also occur [2, 10, 11]. These endocrine dysfunctions not only lead to impaired quality of life and metabolic abnormalities but sometimes induce lethal conditions such as adrenal crisis [10]. Furthermore, these adverse effects can be avoided by stopping or reducing opioid treatment or hormone replacement therapy. However, unfortunately, these opioid-induced endocrine dysfunctions are not widely recognized [2–7]. In this paper, we present a 42-year-old male patient suffering from hypogonadotropic hypogonadism caused by opioid treatment for nonmalignant chronic pain, and we discuss the literatures on the effects of opioids on endocrine functions.

## 2. Case Report

A 42-year-old male patient suffered from severe pain after left brachial plexus injury by traffic accident since 1999. Because his pain was severe and uncontrolled by regular analgesics such as nonsteroidal anti-inflammatory drugs, anticonvulsants, and buprenorphine, transdermal fentanyl was administered since January in 2011, and its dosage was gradually increased. About 4 months after the treatment with transdermal fentanyl (4.2 mg/day), he felt a general fatigue, loss of libido, and erectile dysfunction for the first time, and he referred to our department for further examinations in October 2011. He had no appreciable past history except for traffic injury in 1999. He had also received diclofenac sodium, gabapentin, pregabalin and clonazepam. On admission, he was 163.9 cm tall and weighed 57.1 kg (body mass index: 21.2 kg/m^2^). His blood pressure was 92/53 mmHg, heart rate was 67/min, and body temperature was 36.4°C. A physical examination demonstrated no significant findings including loss of hircus and pubic hair. Radiography of the thorax, as well as an electrocardiogram, was normal. The results of routine laboratory and urine findings were within normal ranges except for slight anemia. Morning (AM 8:00) endocrine examinations including serum thyroid stimulating hormone (TSH), free triiodothyronine (FT3), free thyroxin (FT4), insulin-like growth factor-1 (IGF-1), prolactin (PRL), adrenocorticotropin (ACTH), cortisol, dehydroepiandrosterone-sulfate (DHEA-S), and urinary cortisol were within normal ranges. On the other hand, serum luteinizing hormone (LH) and follicle stimulating hormone (FSH), total testosterone, and free testosterone were decreased (Table 1). Hormone stimulating tests including thyrotropin-releasing hormone (TRH, 0.5 mg), corticotropin-releasing hormone (CRH, 0.1 mg), and growth hormone-releasing peptide 2 (GHRP-2, 0.1 mg) showed normal reactions of TSH, ACTH, cortisol, and growth hormone (GH), respectively. On the other hand, in LH-releasing hormone (LH-RH, 0.1 mg) stimulating test, FSH reaction was delayed (Table 2). These findings showed that he had hypogonadotropic hypogonadism. Enhanced magnetic resonance imaging (MRI) of the hypothalamus and pituitary gland showed no abnormal findings. He had no past history of traumatic brain injury, cranial irradiation, glucocorticoid treatment, and metabolic syndrome accounting for acquired hypogonadotropic hypogonadism. Furthermore, his hypogonadotropic symptom such as loss of libido and erectile dysfunction appeared after starting treatment with transdermal fentanyl. Taken together, we thought that his hypogonadotropic hypogonadism was most likely caused by chronic use of transdermal fentanyl. However, because his chronic pain was severe and uncontrolled by regular analgesics such as nonsteroidal anti-inflammatory drugs, anticonvulsants, and buprenorphine, his transdermal fentanyl treatment could not be reduced or stopped. Therefore, he is planned to have the hormone replacement therapy.

## 3. Discussion

In this paper, we presented a case of hypogonadotropic hypogonadism caused by opioid treatment for nonmalignant chronic pain. To our best knowledge, our case is the first paper describing endocrine dysfunction by opioid treatment from Japan that has been published in the English literature.

Nonmalignant chronic pain is a frequent condition in the general population and recognized as a common health problem causing social and economic losses for the individuals and society [12, 13]. The majority of patients with nonmalignant chronic pain are treated with physical therapies and/or nonopioid analgesics such as non-steroidal anti-inflammatory drugs and anticonvulsants. However, some patients with nonmalignant chronic pain do not obtain sufficient pain relief by these traditional treatments and need further treatments including opioids. Opioid is extremely effective for severe pains, and its prescription for the treatment of malignant and nonmalignant chronic pain is now increasing in the world [1]. Transdermal fentanyl used in the present case became possible to be prescribed under health insurance for patients with nonmalignant chronic pain from 2010 in Japan. However, long-term opioid treatment including transdermal fentanyl has several side effects such as fatigue, nausea, constipation, emotional disturbances, and respiratory suppression. In addition, it has recently reported that opioid treatment induces endocrine dysfunctions in humans [2–11]. 

 Opioid-induced endocrine dysfunction was firstly reported in male heroin and methadone users with hypogonadism in 1973 [14]. Since then, several studies have shown that decreased sexual function with loss of libido and impotence in males and menstrual disturbances in females are common findings in heroin addicts and low levels of gonadotropins and/or sex hormones in such patients [15–18]. In addition, recent studies have shown that patients with oral, intrathecal, or transdermal opioid treatment for chronic pain have hypogonadism [2–9]. The prevalence of opioid-induced hypogonadism was reported to range from 21% to 96% [2–9, 19, 20]. A small observational study with 54 men taking oral opioid showed that serum-free testosterone and total testosterone were less than normal in 56% and 74%, respectively, and that in the men who had normal erectile function before using opioid, 87% suffered from severe erectile dysfunction or diminished libido after starting opioid therapies [4]. In a study with 47 female patients treated with oral or transdermal opioid for chronic pain, serum LH, FSH, estradiol, and DHEA-S levels were lower in both premenopausal and postmenopausal women than those in controls. Menses had stopped in 52% of patients between ages 30 and 50 years, whereas they did only 20% of controls [6]. In another study with 73 patients treated with intrathecal opioid for chronic pain, serum LH and testosterone levels in male patients and serum LH and estradiol in female patients were significantly lower compared to controls. Of male patients, 96% suffered from impotence or decreased libido, and all female patients developed amenorrhea or irregularities in menstruation [2]. One recent study has also reported that opioid-induced hypogonadism is more often observed in men than in women [7]. In addition, some studies have shown dose-response effects on opioid-induced hypogonadism and the improvement of hypogonadism by stopping or reducing opioids [6, 19, 21]. Furthermore, a small prospective study has shown that intrathecal opioid administration resulted in a significant reduction in serum testosterone at one week [3]. However, most of these studies are cross-sectional surveys with small number subjects and with different kinds, dosages, routes, and periods of opioid treatment. Therefore, further large prospective studies are needed to clarify the clinical details such as the prevalence and extent of opioid-induced hypogonadism.

Several mechanisms are suggested to explain opioid-induced hypogonadism. Both endogenous and exogenous opioids, can bind opioid receptors primarily in the hypothalamus [22]. Opioids have been shown to decrease the release of gonadotropin-releasing hormone (GnRH) and disrupt its normal pulsatility in the hypothalamus, resulting in a reduction of the release of LH and FSH from the pituitary gland and that of testosterone and estradiol from the gonads [3, 14, 23–29]. Direct effects of opioids on the pituitary gland and gonads have also been suggested [30–32]. Therefore, patients with opioid-induced hypogonadism have low serum testosterone/estradiol with normal or low serum LH/FSH levels as shown in the present case. Some studies evaluating the effect of opioid on the endocrine system in humans have shown that they have high serum prolactin levels leading to inhibit the secretion of GnRH [9, 33, 34]. However, most of the opioid treatment has no effect on serum prolactin levels as shown in the present case [2, 33, 35, 36]. Furthermore, opioids have also been shown to decrease adrenal androgen, an important precursor of both testosterone in men and estradiol in women [37]. However, the present case has no decreased adrenal androgen such as DHEA-S. 

It is known that there are a variety of symptoms and signs related to hypogonadism such as loss of libido, fatigue, muscle weakness, depressive state, osteoporosis in men and women, erectile dysfunction in men, and menstrual irregularities in women [38]. In the present case, he had erectile dysfunction, loss of libido, and fatigue but not osteoporosis and impaired psychological conditions. Interestingly, several studies have shown the association of hypogonadism with pain. Hypogonadism increases the sensitivity of pain, and sex hormone administration decreases its sensitivity in experimental animals [39–41]. In addition, in morphine-induced hypogonadic male patients with chronic pain, hormone replacement therapy progressively improves pain assessed by a validated Italian pain questionnaire [8]. 

Opioids have also been reported to affect other endocrine functions. Some studies have shown that opioids inhibit hypothalamus-pituitary-adrenal axis [2, 42, 43]. Interestingly, one patient has been reported to develop an adrenal crisis [10]. Another study has also shown that 15% of patients develop GH deficiency [2]. On the other hand, opioids do not seem to affect thyroid function [9, 33]. The present case has no abnormal adrenal, GH, and thyroid functions. 

Although there is no current standard for the management of opioid-induced hypogonadism, some regimens are advocated [36]. The first option is to reduce or stop opioids and to switch to the treatment such as nonopioid pharmacologic agents, physical therapy, and transcutaneous electrical nerve stimulation. The second option is to substitute opioids with buprenorphine, a partial opioid agonist. It has been reported that buprenorphine has lower incidence of hypogonadism compared to other opioids [17, 44]. If opioid treatment cannot be avoided and these techniques are not adequate, it should be considered to start hormone replacement therapy (testosterone in men, and estrogen with or without progesterone in women) to relieve and/or prevent hypogonadism leading to abnormal physical and psychological conditions. In the present case, because his chronic pain was severe and uncontrolled by regular analgesics, physical therapy, and buprenorphine, opioid treatment could not be reduced or stopped. Therefore, he is planned to have the hormone replacement therapy.

In conclusion, the prescription of opioid for the treatment of nonmalignant chronic pain is expected to be increased in the future. It should be considered to do a routine screening for the manifestations and hormonal investigations related to endocrine dysfunctions in patients with long term-opioid treatment.

## Figures and Tables

**Table 1:** Endocrine examinations on admission.

TSH	1.7	*μ*U/mL	(0.4–3.8)
FT3	2.1	pg/mL	(2.0–3.4)
FT4	1.2	ng/dL	(0.9–1.6)
IGF-1	201.0	ng/mL	(94–261)*
ACTH	28.0	pg/mL	(0–60)
Cortisol	11.2	*μ*g/dL	(4.5–24.5)
DHEA-S	99.4	*μ*g/dL	(41.3–218.2)
Urinary cortisol	43.0	*μ*g/day	(10–100)
LH	1.1	mIU/mL	(1.7–11.2)
FSH	1.1	mIU/mL	(2.1–18.6)
Total testosterone	1.0	ng/mL	(2.7–10.7)
Free testosterone	4.5	pg/mL	(7.7–21.6)

( ): normal range.

*Age-specific normal range.

**Table 2:** Hormone stimulating tests on admission.

Time (min)	0	30	60	90	120
(1) TRH, CRH, LH-RH stimulating tests					
TSH (*μ*U/mL)	2.41	8.77	5.25	4.33	3.6
ACTH (pg/mL)	30	69	64	44	42
Cortisol (*μ*g/dL)	13.0	19.1	16.9	15.9	15.1
LH (mIU/mL)	1.1	9.5	8.6	8.3	6.8
FSH (mIU/mL)	0.9	2.0	2.0	2.5	2.2

Time (min)	0	15	30	45	60

(2) GHRP-2 stimulating test					
GH (ng/mL)	<0.05	14.84	23.6	21.59	15.35
